# Role of Gap Junctions and Hemichannels in Parasitic Infections

**DOI:** 10.1155/2013/589130

**Published:** 2013-10-23

**Authors:** José Luis Vega, Mario Subiabre, Felipe Figueroa, Kurt Alex Schalper, Luis Osorio, Jorge González, Juan Carlos Sáez

**Affiliations:** ^1^Departamento de Fisiología, Pontificia Universidad Católica de Chile, 8330025 Santiago, Chile; ^2^Laboratorio de Fisiología Experimental (EPhyL), Instituto Antofagasta (IA), Universidad de Antofagasta, 1270300 Antofagasta, Chile; ^3^Inmunología, Departamento de Tecnología Médica, Universidad de Antofagasta, 1270300 Antofagasta, Chile; ^4^Department of Pathology, Yale School of Medicine, New Haven, CT 06520-8023, USA; ^5^Unidad de Parasitología Molecular, Facultad Ciencias de la Salud, Universidad de Antofagasta, 1270300 Antofagasta, Chile

## Abstract

In vertebrates, connexins (Cxs) and pannexins (Panxs) are proteins that form gap junction channels and/or hemichannels located at cell-cell interfaces and cell surface, respectively. Similar channel types are formed by innexins in invertebrate cells. These channels serve as pathways for cellular communication that coordinate diverse physiologic processes. However, it is known that many acquired and inherited diseases deregulate Cx and/or Panx channels, condition that frequently worsens the pathological state of vertebrates. Recent evidences suggest that Cx and/or Panx hemichannels play a relevant role in bacterial and viral infections. Nonetheless, little is known about the role of Cx- and Panx-based channels in parasitic infections of vertebrates. In this review, available data on changes in Cx and gap junction channel changes induced by parasitic infections are summarized. Additionally, we describe recent findings that suggest possible roles of hemichannels in parasitic infections. Finally, the possibility of new therapeutic designs based on hemichannel blokers is presented.

## 1. Introduction

Members of gap junction (GJ) familiy proteins form intercellular communication channels, which connect the cytoplasm of neighboring cells and hemichannels, which connect the intra- and extracellular milieu [1]. Both intercellular channels and hemichannels participate in physiologic and pathologic processes including electrical conduction [2], inflammation [3], immune system activation [4], tissue repair/remodeling [5], and response to bacterial [6, 7] and viral infections [8]. However, little is known about the role of GJ channels in parasite infection and studies on the possible role of hemichannels are not available. Herein, we summarize the available data on the role of GJ channels in parasitic diseases and we also present new data suggesting that hemichannels might serve as key paracrine communication pathway during parasitic infections.

## 2. Gap Junction Channels and Hemichannels

**Figure 1:**
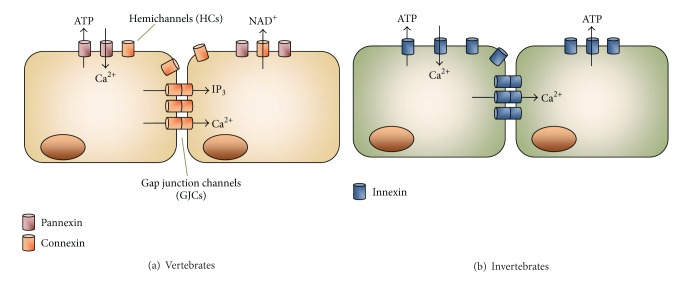
Diagram illustrating gap junction channels and hemichannels. The head to head docking of two hemichannels forms a gap junction channel. Each hemichannel is oligohexamer of the protein subunits, pannexins (Panxs) or connexins (Cxs). (a) In vertebrates, Cxs form hemichannels and gap junction channels, whereas Panxs are believed to form only hemichannels. (b) Invertebrates express innexins and they can form hemichannels and gap junction channels. Hemichannels are permeable to ions including Ca^2+^, and small molecules including signaling ones such as ATP and NAD^+^. Similarly, gap junction channels are permeable to ions and small molecules including IP_3_.

Connexins (Cxs) and pannexins (Panxs) are members of two different GJ protein families in vertebrates [1]. Both protein subtypes can form channels that serve as pathways of cellular communication [1, 9]. Cxs and Panxs show similar membrane topology but only modest sequence homology [1]. In rodents and humans Cxs are encoded by 20 and 21 genes, respectively [10], whereas Panxs include only three members [11]. Moreover, innexins (Inxs) are members of a GJ family expressed only in invertebrates (Figure 1) [12]. They show similar membrane topology with Cxs and Panxs and can also form intercellular channels and hemichannels [13]. Inxs were originally identified in *Drosophila melanogaster *and *Caenorhabditis elegans*; however, Inx genes have been cloned recently from several other invertebrates (reviewed in [13]).

Gap junction plaques are clusters of intercellular channels each one formed by head-to-head docking of two hemichannels (Figure 1) [1]. One hemichannel may dock with another one of identical Cx composition (of the adjacent cell) to form a homotypic gap junction channel or with hemichannels containing different Cx types or Cx composition to form a heterotypic gap junction channel [14]. The latter, provide a direct pathway for diffusional transfer of ions, metabolites and signaling molecules between adjacent cells [1, 9]. These channels are essential in several physiological tissue functions such as cardiomyocyte electrical conduction [2], development and regeneration of skeletal muscle [15], endocrine gland secretion [16], and ovarian folliculogenesis [17]. They are also implicated in pathophysiological conditions including hereditary deafness [18], cataract [19], ectodermal dysplasias [20], tumorigenesis [21], and inflammatory responses [3]. For comprehensive description of some of the aforementioned processes, we refer the readers to several recent reviews [22–25].

Hemichannels serve as autocrine/paracrine cellular communication pathways (Figure 1) [1, 26, 27]. They are permeable to ions and small molecules including cytosolic signals. For example, hemichannels formed by Cxs and Panxs are permeable to adenosine triphosphate (ATP). In addition, Cx hemichannels are permeable to nicotinamide adenine dinucleotide (NAD^+^), glutamate, and prostaglandins [26, 27]. Recently, several studies have shown the relevance of hemichannels in physiological and pathophysiological conditions. The former include cell volume regulation [28], vascular tone [29], T cell activation [4], inflammasome activation [30], proliferation of fibroblasts [31] and neuronal progenitors of the retina [32], ischemic preconditioning of cardiomyocytes [33], ischemic tolerance of neurons [34], neuroglial paracrine interactions [35], and potentiation of skeletal muscle contraction [36]. Among pathological conditions, increased opening of astroglial hemichannels potentiates glutamate-induced neurotoxicity by proinflammatory cytokines [37, 38] and A*β* peptide [39]. In the heart, increased hemichannel activity promotes myocardial damage induced by ischemia [40–43]. For more details about hemichannels, see recent reviews [27, 44–47].

## 3. Effects of Parasite Infections on Gap Junction Proteins

Parasitic infections are a global public health problem in most countries of Asia, Africa, and Latin America, affecting millions of people [48]. Although parasitic diseases have traditionally impacted restricted areas clustered to less economically developed societies and tropical geographic locations, they have gained more attention due to increasing international travels, wide expansion of immune suppressed patients (e.g., HIV-infection, transplant patients, and anticancer treatments), or influx of immigrants from endemic zone to developed countries [48]. Despite the marked increase in their clinical relevance, no vaccines are available for primary prevention and current chemotherapy is associated with considerable toxicity and side effects [48].

 Most studies describing the participation of the Cx-formed gap junctions in parasite infections are related to protozoan parasites such *Trypanosoma cruzi* (*T. cruzi*) or *Toxoplasma gondii *(*T. gondii,* Table 1) [49–55]. Although some studies describe the presence of gap junction channels in parasitic flatworms, the functional significance remains to be elucidated [56–59].

### 3.1. *Trypanosoma cruzi*

*T. cruzi* is the causative agent of Chagas disease that affects about 18 million people in America [60]. The chronic disease causes colopathy, severe arrhythmia, and other electrical heart defects [49–51]. Pioneering studies demonstrated that infection with Tulahuen strain of *T. cruzi* reduces GJ mediated communication in cultured neonatal rat cardiomyocytes [51]. In these cells, *Trypanosoma* infection reduced both junctional conductance and intercellular transfer of the fluorescent dye Lucifer yellow (LY) [51]. Also, levels of Cx43 were significantly reduced at junctional membrane regions [50, 51]. In cultured mouse myocytes, the infection with the Y strain of *T. cruzi* increased Cx43 protein levels at one hour after infection, whereas Cx43 transcripts were unaltered. However, analysis at 24–72 hours post infection showed a decrease in both Cx43 protein (61%) and mRNA (20%) levels in both cultured cells and myocardium of infected mice (~26% reduction of Cx43 protein) [49]. Interestingly, cells with pronounced decrease in Cx43 protein levels showed more abundant intracellular amastigotes, suggesting a direct relationship between host cell parasitism and Cx43 downregulation *in vitro* [49]. In astrocytes and leptomeningeal cell cultures, the infection with Tulahuen strain of *T*. *cruzi* reduced dye coupling between cells, without changes in expression and phosphorylation state of Cx43 [50]. This effect was associated with reduced Cx43 levels in astrocytes and reduced Cx43 and Cx26 levels in leptomeningeal cells [50]. The authors suggested that impaired intercellular communication resulted from altered targeting of Cx protein to the plasma membrane and/or changes in assembly of hemichannels [50].

 Examination of whole brain samples from mice infected with Brazil strain showed reduced Cx43 immunostaining [50]. In addition, mice infected with Y or Brazil *T. cruzi* strains showed a reduction in Cx43 levels in atria and ventricle after 11 or 30 days after infection, respectively [49]. Gene profiling of *T*. *cruzi*-infected cardiomyocytes revealed suppression of *GJA1* and *GJC1* genes, which encode for Cx43 and Cx45 proteins, respectively, at 48 hours after infection [61]. Upregulation of *GJA4 *gene encoding Cx37, a major endothelial cell Cx was also described [62]. Also, samples from chagasic patients showed alterations of GJs in the heart [54]. Immunohistochemical analysis of left ventricle biopsies from subjects with chronic chagasic disease showed reduction in both mean number (~20%) and size (~2.2 fold) of Cx43 plaques [54]. The mechanism of Cx43 reduction after *T. cruzi*  infection is not completely elucidated. However, the participation of CC-chemokines and cytokines such as TGF-*β* and TNF-*α* signaling and perforin-dependent cytolytic mechanisms have been reported [52–55]. In cardiomyocytes, SB-431542, an inhibitor of TGF-*β* receptor type I (ALK-5), reversed the *T. cruzi*-induced Cx43 reduction [54]. Also, treatment with Met-RANTES, a selective CCR1/CCR5 antagonist, significantly ameliorated Cx43 loss in the heart of *T. cruzi*-infected mice [52]. Similarly, murine knockout models have identified TGF-*β* and TNF-*α* signaling in gap junctional alterations observed in *T. cruzi* infection. For example, Cx43 reduction was not observed in TNF-*α* receptor 1 null- or anti-TNF-*α*-treated mice infected with Colombian strain of *T. cruzi* [55]. Similarly, Cx43 expression was unaffected in perforin-deficient mice infected with the Y strain of *T. cruzi* [53].

### 3.2. *Toxoplasma gondii*

Central nervous system toxoplasmosis is a frequent and life-threatening opportunistic infection in immune compromised subjects and is characterized by encephalitis and development of focal brain lesions (e.g., abscess) [63]. Infection by *T. gondii* caused severe GJ alterations in cells of the central nervous system [50]. Infection with ME49 strain of *T. gondii* decreased the intercellular diffusion of LY in both primary cultured leptomeningeal cells and astrocytes [50]. However, no changes in expression or phosphorylation state of Cx43 were observed [50]. Staining of cultured astrocytes and leptomeningeal cells showed that Cx43 immunoreactivity disappeared from appositional membrane areas and Cx26 immunoreactivity was significantly reduced only in parasitized cells. Cx43 and Cx26 normal distribution pattern was maintained in areas of the culture where cells were not parasitized, suggesting a close association between presence of the parasite and impaired intercellular communication [50]. Moreover, brain preparations from mice infected with the ME49 strain of *T. gondii*, showed complete absence of Cx43 immunoreactivity within the cysts and marked reduction in surrounding tissue [50]. Although little is known about the possible influence of *Toxoplasma* infection on the expression of other Cxs and Panxs, the current data support the notion of impaired intercellular communication between central nervous system cells with possible impact in tissue homeostasis and normal function.

### 3.3. *Schistosoma mansoni*

Schistosomiasis is the second most common parasitic infection of humans after malaria [64]. Various strains such as* Schistosoma japonicum*, *Schistosoma mansoni*, *and Schistosoma haematobium* can induce inflammatory conditions in diverse organs including liver, lung, skin, brain, placenta, gastrointestinal and genitourinary tracts [64]. 

 In mice models, it has been shown that Cx43 plays a key role in the formation of hepatic granulomas induced by *Schistosoma mansoni* (*S. mansoni*) [65]. Schistosomiasis infection occurs through the skin during contact with a cercariae released by the intermediate hosts generally amphibious snail species [64]. The host reacts to eggs and egg products by inducing a Th2-mediated immune response may lead to hepatic granulomatous inflammation and pathological tissue remodeling leading to fibrosis [66]. Interestingly, hepatic granulomas induced by *S. mansoni* in Cx43 deficient mice present a higher degree of fibrosis and a reduced index of cell proliferation at 8 and 12 weeks after infection [65]. However, no differences in the average area of granulomas or number of cells per granuloma were observed [65]. The authors suggested that deletion of one allele of Cx43 gene could be the cause of reduced GJ channels that modifies the interactions between granuloma cells, thereby modifying the characteristics of granuloma [65].

## 4. Gap Junction Proteins in Parasites

### 4.1. Functional Evidence

Ultrastructural identification of GJs has been obtained in flatworms such as *Hymenolepis diminuta *[58], *Diphyllobothrium dendriticum* [57], and *Taenia solium *[59, 67]. Also, the existence of channels reminiscent of GJs between protozoan *Trypanosoma musculi* and fibroblast has been described [68]. In invertebrates, both GJ channels and hemichannels are formed by Inx proteins [13].

 Electron microscopy studies revealed a large number of GJs in the neck and immature proglottids of adult *Taenia solium *[67]. A large number of Inx/Panx epitopes have been identified in the parenchyma and tegumentary surfaces in both larvae and adults of *T. solium*. Although it has not been possible to obtain the coding sequences by PCR, the existence of these proteins in flatworms has been demonstrated by western blot analyses [67]. *T. solium strobilae *showed higher uptake of LY when exposed to elevated glucose [69]. The authors reported that they were unable to determine whether these structures correspond to hemichannels or GJ channels. This result suggests that *T. solium *also obtains nutrients directly from the mucosal wall, along the whole strobilar tegument [69]. The identification of hemichannel activity and characterization of their permeability properties in *T. solium* strobilae could be of great relevance for the development of new pharmacological tools.

 Moreover, one study suggests the existence of channels reminiscent of GJ channels between *Trypanosoma musculi*, a parasite specific to mouse, and fibroblasts. This is because LY injected into fibroblast was incorporated into associated trypanosomes [68]. However, the presence of a direct dye transfer pathway has not been demonstrated and might result from dye release to the extracellular space via hemichannels and reuptake also via hemichannels.

### 4.2. Molecular Evidence

Because some parasite genomes have been sequenced, we searched into the genomic databases for the existence of Inx orthologs and paralogs. The search was performed using the “identify genes by protein motif pattern” option, using as reference the motif YYQW of Inx/Panx or simply using the word innexin. The databases used were GeneDB [70], PlasmoDB [71], and TriTrypDB [72]. The result showed the existence of 195 putative Inx-like genes of which 129 were for *Platyhelminths*, 11 for *Nematodes*, 8 for *Plasmodium*, and 47 for *Kinetoplastids*. However, no Inx conserved domains were detected in *Plasmodium* and *Kinetoplastids* sequences. This suggests that protozoa have no Inx orthologs. Inx genes identified in *Platyhelminths* were 24 for *Taenia solium*, 25 for *Schistosoma mansoni*, 24 for *Schistosoma japonicum*, 17 for *Hymenolepis microstoma*, 19 for *Echinococcus multilocularis*, and 20 for *Echinococcus granulosus* (Table 2, Figure 4). We also investigated putative genes in arthropods with relevance in parasitology, and we found 5 for *Pediculus humanus*, 7 for *Anopheles gambiae*, 4 for *Anopheles darlingi*, and 6 for *Aedes aegypti* (Table 2).

## 5. Possible Role of Hemichannels in Parasitic Infections

Because the physiological or pathological role of hemichannels has been proposed only during the last decade, studies on their possible involvement in parasitic infections have not been reported. However, recent studies have shown that hemichannels play an important role in bacterial and viral infections. For example, Cx43 hemichannels increase the internalization of *Yersinia enterocolitica* in HeLa cells [73] and Cx26 hemichannels increase the invasion and dissemination of *Shigella flexneri* in epithelial cells [6, 74]. Also, ^10^Panx1 a peptide that block Panx1 hemichannels inhibited human immunodeficiency virus (HIV) invasion and replication in CD4^+^ T lymphocytes [8]. Although the role of hemichannels in parasitic infections has not been studied, they could participate in responses that include changes in plasma membrane permeability, purinergic or calcium signaling and inflammasome activation. Here, we discuss the possible participation of hemichannels in these cellular processes. 

### 5.1. Host Plasma Membrane Permeability

A common condition and often necessary for infection is the alteration of the host cell membrane permeability [75, 76], and hemichannel activity can considerably affect the permeability of the cell membrane in mammalian cells [47].

*T. cruzi* alters the plasma membrane permeability in host cells during different stages of the disease [76–79]. Infection with *T. cruzi* reduces junctional conductance between infected cells, as determined by voltage-clamp in cardiomyocyte cell pairs [49]. Moreover, in a chronic model of *T.cruzi*-induced myocarditis, increased membrane permeability was detected through the intense ruthenium red labeling predominantly of the subplasmalemmal zone adjacent to the adherent macrophage [79]. The existence of viable channels reminiscent of GJs between parasite and fibroblast-associated cells in *T. musculi*, a protozoan parasite specific to mice was also described [68]. Scanning and transmission electron microscopy showed intimate membrane-to-membrane contact between the adherent cells and parasites, and LY transfected into fibroblast could be incorporated into associated trypanosomes in a coculture of *T. musculi* with spleen-derived adherent fibroblasts [68]. Other studies have shown that permeabilization of host cells in *T. cruzi* infections is due to membrane damage, involving Ca^2+^-dependent exocytosis of lysosomes and delivery of acid sphingomyelinase to the outer leaflet of the cell membrane [76].

**Figure 2:**
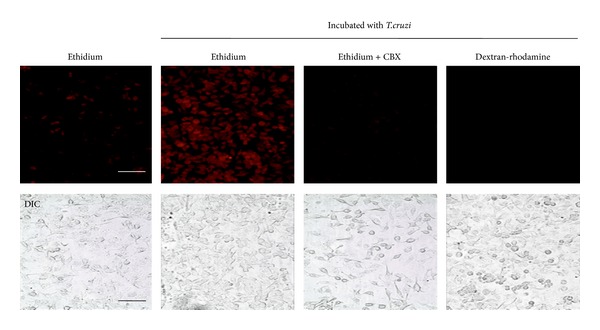
*Trypanosoma cruzi* increases the ethidium uptake in Cx43 HeLa cells. Representative images of ethidium (red) uptake by Cx43-HeLa cells exposed to metacyclic trypomastigotes (4 : 1 parasites/cell) of strain CL Brener of *T. cruzi*. In some cases, cells were pretreated with carbenoxolone (100 *μ*M CBX) for 30 min before exposure to *T. cruzi*. Dextran-rhodamine dye (10 Kda) was added to demonstrate plasma membrane damage. Note that dextran-rhodamine did not stain the cells, indicating integrity of the cell membranes. Uninfected cells were used as control. Bar: 50 *μ*m.

**Figure 3:**
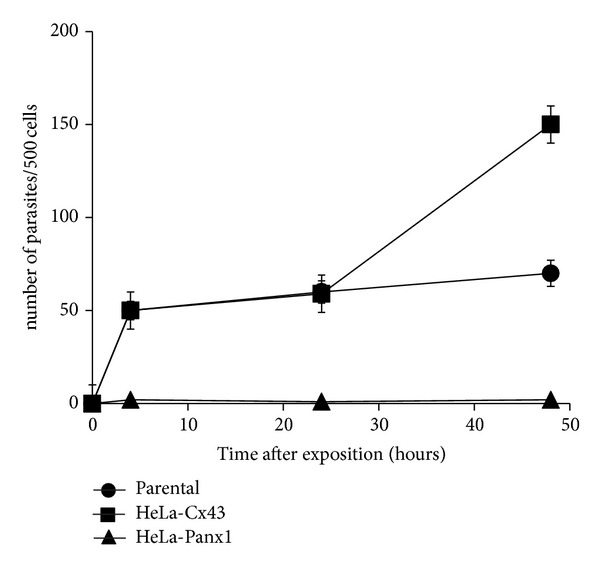
Intracellular *Trypanosoma cruzi* growth depends on type of host hemichannels. HeLa cells transfected with Cx43, or Panx1 were infected with strain Y of *T*. *cruzi*, and the number of intracellular parasites was determined at 4, 24, and 48 hours after infection. The number of parasites per infected cell of a total of 500 infected cells is shown.

**Figure 4:**
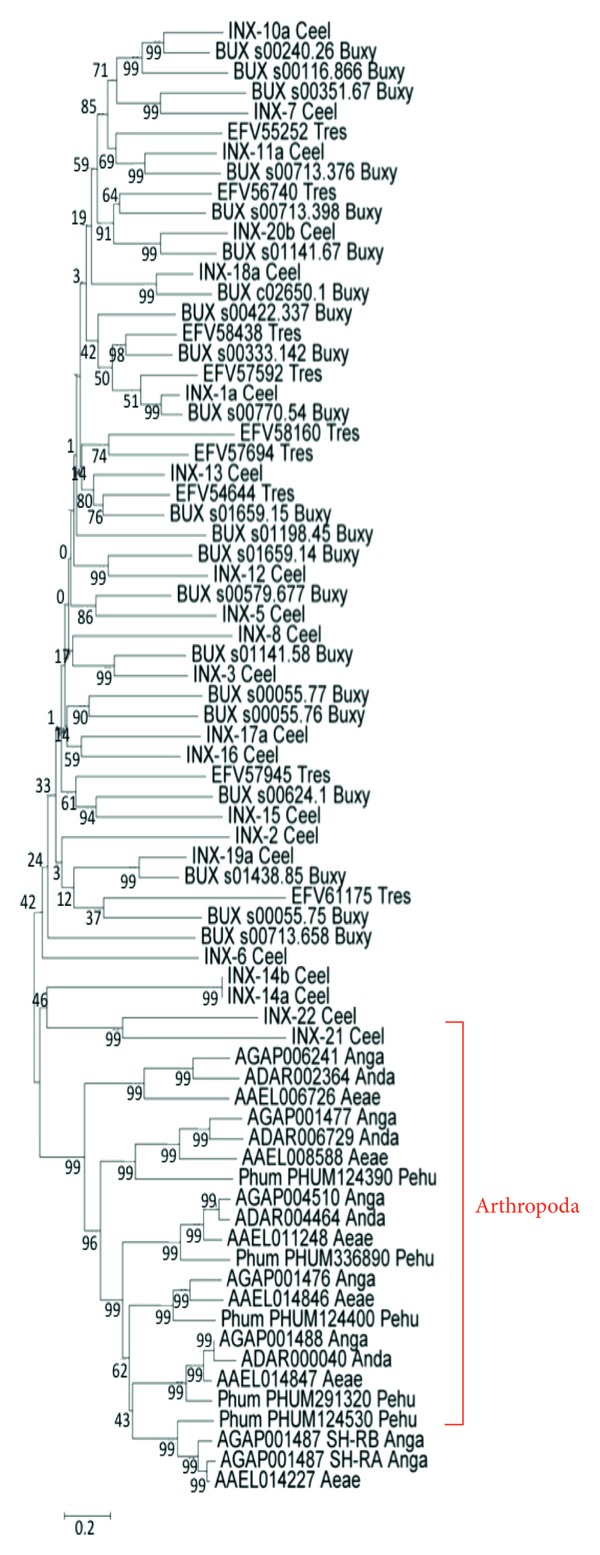
Phylogenetic tree of putative innexin sequences, drawn by the NJ algorithm of the megaprogram. The numbers on the nodes indicate the percent recovery of these nodes in 500 bootstrap replications. Species included are: *Caenorhabditis elegans* (Ceel); *Bursaphelenchus xylophilus* (Buxy); *Trichinella spiralis* (Tres); *Echinococcus granulosus* (Ecgr); *Echinococcus multilocularis* (Ecmu); *Taenia solium* (Taso); *Hymenolepis microstoma* (Hymi); *Schistosoma japonicum* (Scja); *Schistosoma mansoni* (Scma); *Pediculus humanus* (Pehu); *Anopheles gambiae* (Anga); *Anopheles darlingi* (Anda); *Aedes aegypti* (Aeae). The cluster of arthropoda is indicated in red.

 To demonstrate whether hemichannels are involved in parasitic-induced plasma membrane alteration, HeLa cells stably transfected with Cx43 were exposed to metacyclic trypomastigotes of CL Brener strain during 30 minutes. Then, we measured the activity of hemichannels using the ethidium uptake assay (for methodology see [39]). The results revealed that *T. cruzi *increases the ethidium uptake predominantly through hemichannels, since the effect was prominently blocked by 100 *μ*M carbenoxolone, a GJ and hemichannel blocker (Figure 2). Of note, dextran-rhodamine, with a molecular mass of 10 kDa (above the exclusion size limit of hemichannels) did not enter into the cells exposed to *T. cruzi,* suggesting that dye uptake was not a result of membrane breakdown (Figure 2). To evaluate the participation of hemichannels in acute *T. cruzi *infection, we exposed HeLa cells transfected with Cx43 or Panx1 to trypomastigotes during 3 hours, and then cells were washed three times with phosphate buffered saline (PBS) solution and incubated in control medium (DMEM 10% fetal calf serum, 5% CO_2_, 37°C) for additional 48 hours. The numbers of intracellular parasites were then counted at 4 h (immediately after wash), at 24 and 48 hours of incubation. Notably, the number of intracellular parasites (amastigotes) was ~3 times higher in HeLa-Cx43 than in parental HeLa cells at 48 hours (150 ± 5 versus 71 ± 5 parasites per cell (Figure 3)). No differences were observed at 4 or 24 hours. Interestingly, intracellular parasites were not detected in HeLa-Panx1 at any incubation time (Figure 3). These results suggest that hemichannels affect parasite cell invasion and/or proliferation. Studies focusing on the molecular mechanisms are required and could open new avenues on chemotherapy of *T. cruzi* infection.

 Another parasite that alters the plasma membrane permeability is *Plasmodium falciparum*. This parasite invades and replicates asexually within human erythrocytes and enhances plasma membrane permeability in different stages of the disease [80, 81]. The specific channels responsible for those alterations are controversial and currently are termed as new permeability pathways (NPPs) [80, 82, 83]. Some authors have proposed that NPPs are anion-selective channels because malaria-infected erythrocytes exhibit increased anionic channel activity with functional characteristics of a chloride channel, which has been termed plasmodial surface anion channel (PSAC) [82, 84]. The PSAC exhibits a relatively small unitary conductance (20 pS) in molar Cl^−^ solutions and lower open probabilities at positive membrane potentials [85]. It has been estimated that there are 1,000 and 2,000 functional copies per cell in the infected red blood cell membrane [86]. Based on the observation that showed that NPPs are expressed in the infected cell between 10 to 20 hours after infection and their presence was prevented by inhibition of protein synthesis, other authors proposed that NPPs are formed by parasite proteins inserted into the erythrocyte membrane [83, 87]. Recently, two clag3 genes from parasite were described as determinants of the NPP/PSAC channel [88, 89]. The clag3 genes are conserved in all plasmodial species but are absent in other apicomplexan [88]. These genes were identified in blasticidin S-resistant lines of *Plasmodium*, which exhibited reduced expression of clag genes linked to PSAC activity but had no genome-level changes [89, 90]. Silencing of the protein that forms the channel reduced uptake [89]. Interestingly, the silencing affected other clag genes and showed a novel epigenetic resistance mechanism that involves reduction in host cell uptake [89]. Although the biological role of PSAC was unclear, the absence of this channel in other apicomplexan parasites and higher organisms suggest the channel as a potential highly specific therapeutic target [85]. 

 Currently, it is accepted that different uncharacterized channels mediate changes in plasma membrane permeability of infected-erythrocytes, and detailed properties have not yet been identified [83]. Potential candidates to explain these changes are Panx-formed hemichannels, since they are present in erythrocytes, are permeable to molecules such a purines, and exhibit anion-like channel activity when expressed in mammalian cells [91, 92]. The apicomplexan *Babesia divergens* also induces increased erythrocyte permeability [85]. The mechanism for such erythrocyte permeabilization is different in transport rates, solutes selectivity, and temperature dependence compared with the alteration induced by *P. falciparum *[85]. Electrophysiological measurements on *Babesia divergens*-infected cells indicate that PSAC are not present and conductive anion permeability is not increased [85]. Interestingly, [^3^H]-glucose uptake was increased in bovine erythrocytes after *Babesia bovis* infection, and this effect was not mediated by glucose transporters, because the influx of glucose was not saturable at high concentrations and was unaffected by cytochalasin B or phloretin [93]. Interestingly, it was recently found in skeletal muscle that Panx1 hemichannels are permeable to 2-NBDG, a fluorescent glucose derivative and could therefore Panx1 hemichannels could explain changes in glucose uptake in red blood cells infected by parasites [36]. Together, these data support the need to explore the possible role of hemichannels in parasite infections.

### 5.2. Parasitophorous Vacuole Membrane Permeability

Some obligates intracellular parasites, such as *Encephalitozoon*, *Plasmodium*, and *Toxoplasma* live in a parasitophorous vacuole in the host cell [94]. They induce the formation of pores in the parasitophorous vacuole membrane (PVM) that communicate the cytoplasm with the vacuole lumen [94]. 

 Parasites such as *Toxoplasma gondii* and *Plasmodium* contain a sorting signal or motif at the N-termini of proteins that facilitates export of proteins to the host cytosol [95, 96]. This motif, known as the host targeting *Plasmodium* export element (HT/PEXEL), directs *Plasmodium* or *Toxoplasma* proteins into host cells to remodel their cytoskeleton, establishes infection, and promotes parasite survival [95, 96]. In *Toxoplasma*, some proteins that have the motif are not directed to the host cytosol, which indicates a different trafficking mechanism between *Toxoplasma* and *Plasmodium* [97]. *Plasmodium falciparum* exports proteins across the PVM into the erythrocyte cytosol through a protein complex known as *Plasmodium*  translocon of exported proteins (PTEX) [95, 97–99]. This complex is ATP-powered, and comprises heat shock protein 101 (HSP101), PTEX88, PTEX150, exported protein 2 (EXP2), and thioredoxin 2 (TRX2) [97]. Recent studies have also suggested the presence of channel-like translocons with similarities to porins and GJ channels [96, 98, 99]. 

 Assays of microinjection of fluorescent dyes conjugates in the cytosol and subsequent visualization of dye inside the vacuoles have demonstrated the presence of pores in PVM [100]. For example, peptides of 0.8–1.1 or 0.5 kDa dextran could rapidly enter into the parasitophorous vacuole; however, 10 kDa dextran are excluded [100]. This results indicates that the PVM possesses pores with an exclusion size of <10 kDa similar to most studied hemichannels [100]. 

 Patch-clamp studies of the PVM from malaria parasites showed large conductance channels (140 pS). Characterization of these channels showed a high capacity and low affinity molecular sieve, and permeability for soluble macromolecules of >1400 Da [101, 102]. These conductance and permeability properties resemble those of GJ channels and hemichannels.

 In *T. gondii* infection, the microinjection of LY into the cytoplasm of infected fibroblast showed rapid spreading of the dye into the vacuolar space [103]. Similarly, when the dye was injected directly into the vacuolar space, it was later observed in the cytoplasm [103]. Pretreatment of cells with 5 mM probenecid, a Panx1 hemichannel blocker, did not affect the spread of dye into the vacuole, suggesting that Panx1-formed hemichannels are not involved in this phenomenon [103]. Studies focusing on demonstrating the presence of other hemichannels (e.g., Cxs-formed channels) in the PVM could be interesting and relevant to understand the complex host-parasite interaction.

### 5.3. Intracellular Ca^2+^ Mobilization

GJs participate in Ca^2+^ signaling, and they constitute one pathway for intercellular Ca^2+^ wave propagation in cardiomyocytes, astrocytes, and osteocytes, among other cell types [104]. In addition, Cx26, Cx32 and Cx43 hemichannels are permeable to Ca^2+^ [105–108]. Purified Cx43 hemichannels reconstituted into unilamellar liposomes loaded with Calcium Green-2, a Ca^2+^ indicator, showed that an increase between 5 to 20 *μ*M in extraliposomal Ca^2+^ concentration causes a 2-fold increase in Green-2 fluorescence, demonstrating that Cx43 hemichannels are permeable to Ca^2+^ [107]. In protozoan infections, a key process in early stages of invasion is the rise in cytosolic free Ca^2+^ concentration [109]. For example, when *T. cruzi* comes into contact with the host cell, triggers a transient increase in cytosolic free Ca^2+^ concentration that induces host cell lysosome exocytosis [76, 109]. This process is required for cell invasion, because chelating the intracellular Ca^2+^ transients in host cells reduces the entry of the parasite into the cell [110]. In cardiomyocytes, *T. cruzi* infection induces Ca^2+^ fluxes and causes myofibrillar breakdown disturbing contractility [111]. Also, the intracellular free Ca^2+^ concentration is important in *T. gondii* infections. During invasion, the parasite induces a significant increase in cytosolic free Ca^2+^ concentration in phagocytic cells [112, 113]. In dendritic cells, a lysate of *T. gondii* induces an increase of intracellular free Ca^2+^ signal [114]. Moreover, the parasite egress can be artificially induced by the use of ionophores [115, 116]. Since all studied hemichannels are sensitive to Ca^2+^ and also allow passage of Ca^2+^, they could participate in key processes such as invasion in parasite infections of most tissues.

### 5.4. Activation of Inflammasome

The inflammasome has been proposed such a key multiprotein complex involved in innate immunity [30, 117]. Inflammasome activation triggers innate immune defense by inducing the processing of proinflammatory cytokines, such as IL-1, in a caspase 1-dependent manner [117]. Panx1 hemichannels play a key role in inflammasome activation [117]. It has been proposed that small pathogen-associated molecule patterns (PAMPs) can gain cytosolic access via the P2X_7_ receptor/Panx1 hemichannel (P2X_7_/Panx1) complex and activate the inflammasome [117]. It has also been described that ATP release through P2X_7_/Panx1 hemichannels can promote NLR-mediated inflammasome assembly [118, 119]. Recently, some studies have begun to elucidate the role of inflammasome in parasite infection [120]. For example, NALP1 (NACHT-LRR-PYD-containing protein-1)-mediated inflammasome activation is critical for mediating innate immune response to *T. gondii* [120]. NALP1 silencing with siRNA, attenuated the progression of *T. gondii* infection, with accelerated host cell death and eventual cell disintegration. In this study *T. gondii* infection was not observed in monocytic cells with NALP1 knockdown [120]. In addition, malarial hemozoin activates the NLRP3 (NOD-like receptor pyrin domain containing three) inflammasome through Src family kinases [121]. These studies are the first demonstration of the role of inflammasome in parasitic infections. However, the possible relevance of P2X_7_/Panx1-induced inflammasome activation in parasitic infections remains to be determined.

## 6. Conclusions and Perspectives

Parasitic infections affect predominantly underprivileged areas of the world, but attention has been increasing lately due to the rise in people migration habits, intercontinental travels, and immune suppressed patients. Parasitic infections represent serious life threatening conditions in high risk groups such as young children, elderly, and immune deficient subjects, and therapeutic options include a wide variety of compounds with considerable toxicity and side effects. The introduction of new methods, specific inhibitors, and the use of knockout animals has increased our understanding about the role of hemichannels in pathophysiological infectious conditions such as viral and bacterial infections. However, their role in parasitic infections has not yet been explored. Hemichannels are involved in the regulation of plasma membrane permeability in ischemic insults, metabolic inhibition, and cerebral stroke [122, 123]. Under these conditions, the deregulated hemichannel opening increases the cell damage through imbalances in transmembrane electrochemical gradients [35, 123]. Alterations of plasma membrane is a common phenomenon in parasite-induced infections such malaria and *T. cruzi*, among others [81, 124]. In addition, we have shown here for the first time to our knowledge that *T. cruzi* induces opening of Cx43 hemichannels in HeLa cells. Hemichannels could be key players in parasite-induced plasma membrane permeabilization, consequent activation of inflammasome, and cell degeneration. Hemichannels play a key role in Ca^2+^ influx into cells under different conditions such ischemic damage [108]. Moreover, elevations of intracellular Ca^2+^ in the host are required for invasion several parasites that include *T. gondii* and *T. cruzi*, among others [109, 114]. Hemichannels could serve as prominent pathways for Ca^2+^ entry facilitating the above-mentioned processes. Pannexin-1 hemichannels are crucial for inflammasome activation [125]. This cytosolic multiprotein complex is a critical for the innate defense against pathogens [126], suggesting that hemichannels could also be involved in innate defense against parasitic infections. Accordingly hemichannels are regulated by inflammatory cytokines (IL-1*β* and TNF-alpha) [37], and parasitic infections promote inflammatory responses. All the above data support the importance of studying the possible role of hemichannels in parasitic infections. They could be important in the invasion, replication, or pathogenesis, especially in protozoan infection. Moreover, they are potential targets for the development of new compounds to limit parasite infections or tissue/organ damage induced by their presence.

## Figures and Tables

**Table 1:** Summary of published works on the effect of parasite infections on the gap junction activity or connexin expression.

Infectious agent	Strain	Cell type	Effects	Experimental techniques	Ref.
*Trypanosome cruzi *	Tulahuen	Primary culture of rat neonatal cardiomyocytes	**↓** dye coupling	Junctional conductance and dye transfer of Lucifer yellow	[50]
			**↓** Cx43 expression at junctional membrane regions	Immunofluorescence	

*Trypanosome cruzi *	Tulahuen	Primary culture of rat neonatal astrocytes	↓ dye coupling	Dye transfer of Lucifer yellow	[49]
			= Cx43 expression at 72 hours p.i.	Western blot (Cx43)	
			= Cx43 phosphorylation state at 72 hours p.i.	Western blot (Cx43) and electrophoretic mobility	
			↓ Cx43 immunoreactivity	Immunostaining (Cx43)	
		Primary culture of rat leptomeningeal cells	↓ dye coupling	Dye transfer of Lucifer yellow	
			= Cx43 expression at 72 hours p.i.	Western blot (Cx43)	
			= Cx43 phosphorylation state at 72 hours p.i.	Western blot (Cx43) and electrophoretic mobility	
			↓ Cx43 and Cx26 immunoreactivity	Immunostaining (Cx43, Cx26)	
	Brazil	Murine infection	↓ Cx43 expression at 4 weeks p.i.	Immunofluorescence	

*Trypanosome cruzi *	Y	Primary culture of mouse neonatal cardiomyocytes	**↑** Cx43 expression and phosphorylation at 1 hour p.i.	Western blot (Cx43)	[50]
			↓ Cx43 expression at 48 and 72 hours p.i.	Immunostaining (Cx43)	
		Murine infection	↓ Cx43 in atria and ventricle at 11 days p.i.	Confocal microscopy (Cx43)
			↓ Cx43 plaque distribution	

*Trypanosome cruzi *	Colombian	C57BL/6 mice	↓ Cx43 expression at 28 and 32 days p.i.	Immunohistochemistry (Cx43)	[54]
		B6.129-Tnfraf1a (p55/60)-deficient mice	= Cx43 expression at 28 and 32 days p.i.		

*Trypanosome cruzi *	Colombian	C3H/He (H-2K) mice	↓ Cx43 expression at 150 and 180 days p.i.	Immunohistochemistry (Cx43)	[51]
		Met-RANTES-treated C3H/He (H-2K) mice	= Cx43 expression at 150 and 180 days p.i.		

*Trypanosome cruzi *	Y	Primary culture of mouse neonatal cardiomyocytes	↓ Cx43 expression at 48 hours p.i.	Immunohistochemistry (Cx43)	[53]
		Left ventricle fragments from the heart of CCC patients	↓ Cx43 plaque number		
			↓ Cx43 plaque size		

*Trypanosome cruzi *	Colombian	Primary culture of mouse neonatal cardiomyocytes	↓ Cx43 gene	Microarray assay	[61]
			↑ Cx37 gene		
			= Cx40 and Cx45 gene		

*Toxoplasma gondii *	ME49	Primary culture of rat neonatal astrocytes	↓ dye coupling	Dye transfer of Lucifer yellow	[49]
			= Cx43 expression at 72 hours p.i.	Western blot (Cx43)	
			= Cx43 phosphorylation state at 72 hours p.i.	Western blot (Cx43) and electrophoretic mobility	
			↓ Cx43 immunoreactivity	Immunostaining (Cx43)	
		Primary culture of rat leptomeningeal cells	↓ dye coupling	Dye transfer of Lucifer yellow	
			= Cx43 expression at 72 hours p.i.	Western blot (Cx43)	
			= Cx43 phosphorylation state at 72 hours p.i.	Western blot (Cx43) and electrophoretic mobility	
			↓ Cx43 and Cx26 immunoreactivity	Immunostaining (Cx43, Cx26)	
		Murine infection	↓ Cx43 expression at 12 weeks p.i.	Immunofluorescence	

**Table 2:** Innexin putative genes in platyhelminths, nematode, and arthropoda.

Organism	Clinical relevance	Gene ID	Description
Platyhelminths			
*Taenia solium *	Neurocysticercosis	TsM_000900900	Innexin
		TsM_000901000	Innexin unc 7
		TsM_000464200	Innexin unc 9
		TsM_000464300	Innexin unc 9
		TsM_000569100	Innexin unc 9
		TsM_000569300	Innexin unc 9
		TsM_000557300	Innexin unc 9
		TsM_000954500	Innexin unc 9
		TsM_000101700	Innexin unc 7
		TsM_001009500	Innexin unc 7
		TsM_001009600	Innexin
		TsM_000471300	Innexin
		TsM_000116500	Innexin
		TsM_000916100	Innexin unc 9
		TsM_000916200	Innexin
		TsM_000028400	Innexin
		TsM_000349500	Innexin unc 9
		TsM_000832100	Innexin unc 9
		TsM_000811500	Innexin unc 9
		TsM_001199700	Innexin unc 9
		TsM_000337000	Innexin
		TsM_000405800	Innexin unc
		TsM_000655000	Innexin unc
		TsM_000883500	Innexin unc 9

*Schistosoma mansoni *	Schistosomiasis	Smp_058470	Innexin unc 7
		Smp_187190	Innexin unc 9
		Smp_141390	Innexin unc 9
		Smp_141290	Innexin
		Smp_141290	Innexin
		Smp_141290	Innexin
		Smp_141290	Innexin
		Smp_140850	Innexin unc 9
		Smp_140860	Innexin unc 9
		Smp_034610	Innexin
		Smp_200490	Innexin
		Smp_129020	Innexin unc 9
		Smp_142390	Innexin unc 9
		Smp_037510	Innexin unc 9
		Smp_146940	Innexin
		Smp_105760	Innexin
		Smp_066900	Innexin unc 9
		Smp_170070	Innexin
		Smp_088060	Innexin unc
		Smp_117170	Innexin
		Smp_161890	Innexin
		Smp_073360	Innexin unc 9
		Smp_161900	Innexin unc 9
		Smp_073380	Innexin unc 9
		Smp_026570	Innexin

*Schistosoma japonicum *	Schistosomiasis	Sjp_0059220	Innexin-5
		Sjp_0088670	Innexin unc 7
		Sjp_0088360	Innexin unc 9
		Sjp_0090890	Innexin unc 9
		Sjp_0111560	Innexin unc 9
		Sjp_0098040	Innexin unc 9
		Sjp_0101240	Innexin unc 9
		Sjp_0114320	Innexin unc 7
		Sjp_0073220	Innexin-10
		Sjp_0073210	Innexin unc 7
		Sjp_0076990	Innexin unc 9
		Sjp_0103760	Innexin unc 9
		Sjp_0131850	Innexin unc 9
		Sjp_0104570	Innexin unc 9
		Sjp_0112260	Innexin unc 7
		Sjp_0111510	Innexin-5
		Sjp_0057750	Innexin unc 7
		Sjp_0094620	Innexin unc 7
		Sjp_0050370	Innexin unc 7
		Sjp_0078510	Innexin unc 9
		Sjp_0056570	Innexin unc 9
		Sjp_0056580	Innexin
		Sjp_0056560	Innexin unc 9
		Sjp_0038790	Innexin unc 9

*Hymenolepis microstoma *	Hymenolepiasis^1^	HmN_000939600	Innexin unc 9
		HmN_000939500	Innexin unc 9
		HmN_000878800	Innexin unc 9
		HmN_000680500	Innexin unc 9
		HmN_000680700	Innexin 1
		HmN_000210900	Innexin unc 9
		HmN_000053700	Innexin
		HmN_000555900	Innexin unc 9
		HmN_000279100	Innexin unc 9
		HmN_000279200	Innexin unc 9
		HmN_000602700	Innexin unc 7
		HmN_000749400	Innexin unc 9
		HmN_000749500	Innexin unc 9
		HmN_000749600	Innexin unc 9
		HmN_000635700	Innexin unc 9
		HmN_000635800	Innexin
		HmN_000143000	Innexin unc 7

*Echinococcus multilocularis *	Alveolar echinococcosis	EmuJ_000628300	Innexin unc 7
		EmuJ_000628400	Innexin
		EmuJ_000527700	Innexin unc 7
		EmuJ_000448500	Innexin unc 9
		EmuJ_000688600	Innexin
		EmuJ_000688700	Innexin unc 9
		EmuJ_000755500	Innexin unc 9
		EmuJ_000838500	Innexin unc 9
		EmuJ_001000000	Innexin unc 9
		EmuJ_000442800	Innexin unc 7

*Echinococcus multilocularis *	Alveolar echinococcosis	EmuJ_000448600	Innexin unc 9
		EmuJ_000500900	Innexin unc 7
		EmuJ_000501000	Innexin unc 7
		EmuJ_000501100	Innexin unc 9
		EmuJ_000501300	Innexin unc 9
		EmuJ_000231100	Innexin unc 9
		EmuJ_000249300	Innexin
		EmuJ_000249400	Innexin unc 9
		EmuJ_000249500	Innexin unc 9

*Echinococcus granulosus *	Cystic echinococcosis	EgrG_000755500	Innexin unc 9
		EgrG_000249300	Innexin
		EgrG_000249400	Innexin unc 9
		EgrG_000249500	Innexin unc 9
		EgrG_001000000	Innexin unc 9
		EgrG_000628300	Innexin unc 7
		EgrG_000628400	Innexin
		EgrG_000527700	Innexin unc 7
		EgrG_000527800	Innexin
		EgrG_000688600	Innexin
		EgrG_000688700	Innexin unc 9
		EgrG_000231100	Innexin unc 9
		EgrG_000838500	Innexin unc 9
		EgrG_000442800	Innexin unc 7
		EgrG_000448500	Innexin unc 9
		EgrG_000448600	Innexin unc 9
		EgrG_000500900	Innexin unc 9
		EgrG_000501000	Innexin unc 7
		EgrG_000501100	Innexin unc 9
		EgrG_000501300	Innexin unc 9

Nematode			
*Trichinella spiralis *	Trichinosis	EFV61175	n.d.
		EFV58438	n.d.
		EFV58160	n.d.
		EFV57945	n.d.
		EFV57694	n.d.
		EFV57592	n.d.
		EFV56740	n.d.
		EFV55252	n.d.
		EFV54644	n.d.
		EFV52506	n.d.
		EFV51028	n.d.

Arthropoda			
*Pediculus humanus *	Pediculosis	Phum_PHUM336890	Innexin inx2
		Phum_PHUM291320	Innexin inx2
		Phum_PHUM124530	Innexin inx2
		Phum_PHUM124400	Innexin inx1
		Phum_PHUM124390	Innexin inx2

*Anopheles gambiae *	Vector	AGAP001476	n.d.
		AGAP001487 (A)	Innexin shaking-B
		AGAP001487 (B)	Innexin shaking-B
		AGAP006241	n.d.

*Anopheles gambiae *	Vector	AGAP001488	n.d.
		AGAP004510	n.d.
		AGAP001477	n.d.

*Anopheles darlingi *	Vector	ADAR006729	n.d.
		ADAR000040	n.d.
		ADAR004464	n.d.
		ADAR002364	n.d.

*Aedes aegypti *	Vector	AAEL011248	Innexin
		AAEL008588	Innexin
		AAEL014847	Innexin
		AAEL006726	Innexin
		AAEL014227	Innexin shaking-B
		AAEL014846	Innexin

n.d.: not determined; unc: uncoordinated protein; ^1^rodent tapeworm to the genus *Hymenolepis* that rarely infects humans.
